# Exploring the Potential of Sustainable Acid Whey Cheese Supplemented with Apple Pomace and GABA-Producing Indigenous *Lactococcus lactis* Strain

**DOI:** 10.3390/microorganisms11020436

**Published:** 2023-02-09

**Authors:** Justina Mileriene, Loreta Serniene, Beatrice Kasparaviciene, Lina Lauciene, Neringa Kasetiene, Gintare Zakariene, Milda Kersiene, Daiva Leskauskaite, Jonas Viskelis, Yiannis Kourkoutas, Mindaugas Malakauskas

**Affiliations:** 1 Department of Food Safety and Quality, Veterinary Academy, Lithuanian University of Health Sciences, Tilzes St. 18, LT-47181 Kaunas, Lithuania; 2 Department of Food Science and Technology, Kaunas University of Technology, Radvilėnų pl. 19, LT-44249 Kaunas, Lithuania; 3 Laboratory of Biochemistry and Technology, Institute of Horticulture, Lithuanian Research Centre for Agriculture and Forestry, Kauno St. 30, Babtai, LT-54333 Kaunas, Lithuania; 4 Laboratory of Applied Microbiology & Biotechnology, Department of Molecular Biology & Genetics, Democritus University of Thrace, 68100 Alexandroupolis, Greece

**Keywords:** acid whey cheese, apple pomace, probiotic *Lactococcus lactis*, GABA, functional cheese

## Abstract

This study aimed to utilize two by-products, acid whey and apple pomace, as well as an indigenous *Lactococcus lactis* LL16 strain with the probiotic potential to produce a sustainable cheese with functional properties. Acid whey protein cheese was made by thermocoagulation of fresh acid whey and enhancing the final product by adding apple pomace, *L. lactis* LL16 strain, or a mixture of both. The sensory, the physicochemical, the proteolytic, and the microbiological parameters were evaluated during 14 days of refrigerated storage. The supplementation of the cheese with apple pomace affected (*p* ≤ 0.05) the cheese composition (moisture, protein, fat, carbohydrate, and fiber), the texture, the color (lightness, redness, and yellowness), and the overall sensory acceptability. The addition of the presumptive probiotic *L. lactis* LL16 strain decreased (*p* ≤ 0.05) the concentration of glutamic acid, thus increasing γ-aminobutyric acid (GABA) significantly in the acid whey cheese. The supplementation with apple pomace resulted in slightly (*p* < 0.05) higher counts of *L. lactis* LL16 on day seven, suggesting a positive effect of apple pomace components on strain survival. The symbiotic effect of apple pomace and LL16 was noted on proteolysis (pH 4.6-soluble nitrogen and free amino acids) in the cheese on day one, which may have positively influenced the overall sensory acceptance.

## 1. Introduction

Sustainability and consumer demand for nutritious food have been vital factors in the dairy industry in recent years [1]. Responding to the demands of a circular economy, cheese producers are searching for ways to reintroduce acid whey, which is the main by-product of cottage cheese manufacturing, back into production [2], bringing along environmental, health, and economic benefits [3]. The production of curd cheese leaves up to 90% acid whey as a byproduct [4], which is considered to be a waste material by small and medium dairy plants [5]. Due to the lower amounts of whey proteins compared with sweet whey, and its distinguishable sour taste, acid whey is considered to be less valuable. Nevertheless, many valuable nutritional components (lactose, galactose, protein, fat, calcium phosphate, and lactic acid) remaining in this by-product can be converted into nutritional food products or co-products by thermocoagulation [6]. In order to improve the sensory properties of the final product and enhance its functionality supplementation with natural sweeteners, bioactive components, such as prebiotics and probiotics [7,8], should be investigated.

Approximately 14.8% of agro-food waste is generated during the cultivation and the processing of fruits and vegetables. However, plant waste is rich in highly applicable compounds, such as various sugars and indigestible carbohydrates (resistant starch, inulin, cellulose, hemicellulose, pectin, alginates, etc.) [9], which can be further used as natural sweeteners and as a dietary fiber and prebiotics in human nutrition [10]. Apple pomace is an abundant and locally available fruit by-product that is generated from apple (*Malus domestica* Borkh) juice processing. It is a waste product that can cause environmental pollution, and even public health hazards, if it is not properly managed [11]. The dry product contains approx. 11% moisture, 51% dietary fiber [12], 10–15% pectin [13], and 4–5% crude protein [14]. It is safe to be used as human food [15] and is valued for its antioxidant properties, its high phenolic content, and its total flavonoid content [16], which are known for antimicrobial, anticancer, and cardiovascular-protective activities [17].

It has been reported that whey proteins do not adhere to water or lipid particles as effectively as casein during processing of whey cheese [1]; therefore, the supplementation of acid whey cheese with fiber-rich apple pomace may be technologically viable, providing water hydration, fat adsorption, and viscosifying and texturizing properties [18]. In addition, the pectin that is contained in apple products may act as a stabilizer in the gel matrix of such dairy products, reducing the syneresis values [19].

Furthermore, various indigenous, protective, and probiotic-type lactic acid bacteria (LAB) are being widely used as protective cultures, nutraceuticals, and flavor enhancers in dairy produce [20,21]. Due to their capacity to produce acid in milk and to transform milk fat and protein into flavor compounds, *L. lactis* subsp. *lactis* are one of the essential components in starter cultures [20,22]. They may be used as a bio-supplement to produce organic acids, bacteriocins, health-promoting bioactive peptides, and amino acids [22], such as GABA, that stimulate the immune system, prevent inflammatory processes, hypertension, and diabetes, and regulate energy metabolism [23]. By producing GABA during fermentation, LAB protect themselves from various types of stress, such as low pH, changes in osmosis, or lack of nutrients [24]. GABA is naturally present in small amounts in many plant foods, and high concentrations are found in fermented products, especially in fermented dairy products [25]. A few previous studies have shown that some of *L. lactis* subsp. *lactis* strains that were isolated from raw and fermented milk [26,27] or kimchi [28] were resistant to bile salts and acids (pH 2.5–3) and exhibited antimicrobial activity against some pathogens (*Listeria monocytogenes*, *Staphylococcus aureus*, and *Salmonella typhimurium*), classifying them as probiotics. Such strains may require a suitable support for their harboring, their protection, and their delivery [29]. Although it has been reported that apples have been successfully used as immobilization support in several food products [19,30,31], protecting probiotics during harsh food processing conditions, such as during freeze drying [32] and during in vitro gastrointestinal stress tolerance tests [33,34], it is not yet clear if the strain is able to thrive in the presence of powdered apple pomace. Our previous studies have reported acid whey protein as an appropriate matrix for the survival of indigenous *L. lactis* subsp. *Lactis* for up to eight days; however, the effect of the supplementation of such matrix with apple pomace on the survival of the strain has never been investigated. Therefore, the aim of this study was to utilize two by-products of the food industry (acid whey and apple pomace), as well as an indigenous *Lactococcus lactis* LL16 strain with probiotic potential, to produce a novel cheese product with functional properties. Moreover, we aimed to characterize the value-added properties of this cheese by analyzing the GABA contents, the sensory properties, and the physicochemical, proteolytic, and microbiological changes during 14 days of refrigerated storage.

## 2. Materials and Methods

### 2.1. Microorganisms

Before being used in this study, *Lactococcus lactis* subsp. *lactis* LL16 strain (LL16) was isolated from raw cows’ milk [35] and was stored at −80 °C in M17 broth (Merck, Germany) with 30% glycerol. In a previous study by Kondrotiene et al. [26], *L. lactis* LL16 showed probiotic potential by surviving in vitro gastric conditions (with resistance to bile and acid), showed good auto-aggregation activity and cell surface hydrophobicity, and proved to be a fast-acidifying strain, as well as having the ability to form a pleasant aroma during milk fermentation, which indicated that it can be used in the development of functional dairy foods. Regarding antibiotic susceptibility, hemolytic, gelatinase, and enzymatic activities, *L. lactis* LL16 showed no activities of undesirable traits and proved to be safe. Growing the strain in MRS broth (Biolife, Milan, Italy) for 18 h at 30 °C until it reached 8–9 log cfu/mL revitalized the strain. The biomass of *L. lactis* with a concentration of 10 log cfu/g was collected by centrifugation (4000× *g* rpm, 15 min, 4 °C).

### 2.2. Materials

The fresh acid whey (6.40% dry matter, 0.35% fat, 0.90% protein, 3.83% lactose, and 5.04 pH, determined with a LactoScan milk analyzer (Bulgaria), according to manufacturer’s instructions) was obtained from a local cheese factory in Lithuania as a by-product of traditional sour curd production. The acid whey protein was produced in the same factory immediately after the collection of the acid whey.

The apple pomace (AP) was obtained from the local apple juice plant (Paslaugos zemdirbiams, UAB, Kauno g. 23, Babtai, Kaunas distr., LT-54334). The pomace was lyophilized in the Laboratory of Biochemistry and Technology at the Institute of Horticulture. The freeze-dried and powdered apple pomace was added to prebiotic-supplemented cheese samples to achieve 3 g per serving size (about 100 g).

### 2.3. Production of Acid Whey Protein Cheese

**Figure 1:**
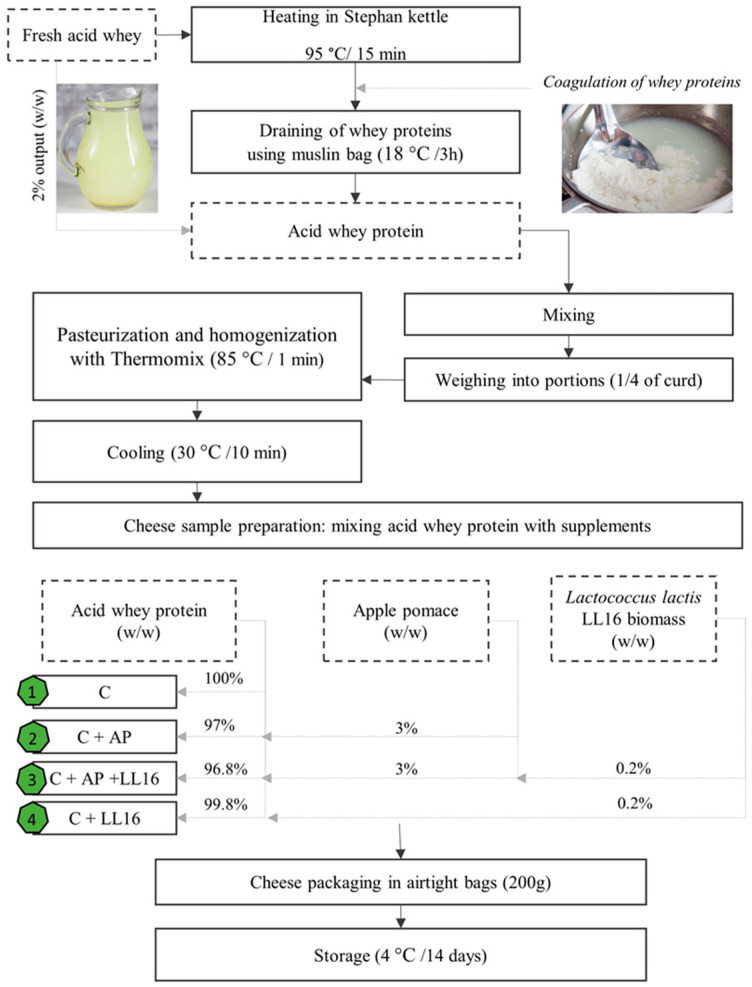
Schematic representation of acid whey protein cheese production.

The acid whey cheese was produced according to traditional whey cheese production methods [36], with some changes, i.e., excluding the acidification of the whey. The production of acid whey protein cheese is schematically depicted in Figure 1. Fresh acid whey was heated in a Stephan kettle (UM/SK 200, Belgium) for 15 min at 95 °C. The coagulated whey protein was then collected in a muslin bag and allowed to drip and cool for 3 h at room temperature (3.5 kg of the acid whey protein was obtained from a kettle with 200 kg acid whey capacity). Then, the curd was removed from the bag, placed into a bowl, thoroughly mixed, and then divided into four equal portions. To lower the risk of contamination from the muslin bags [37], each portion was pasteurized at 85 °C for 1 min, homogenized in Thermomix (Vorwerk, Wuppertal, Germany), and then cooled. Afterwards, the acid whey protein was mixed with apple pomace (3%, *w*/*w*) and/or *L. lactis* LL16 biomass (0.2%, *w*/*w*) to produce 200 g cheese samples (C, C + AP, C + LL16, C + AP + LL16). The acid whey protein cheese samples were packed in sterile hermetic bags and were kept at 4 °C for 14 days. For all following analyses, the samples were analyzed in triplicate.

### 2.4. Microbiological Analysis

Viable counts of LAB, Enterobacteriaceae, coliforms, and fungi were determined in triplicate on the selective media for each species at storage days 1, 7, and 14 using the pour plate technique, as described by Mileriene et al. [37]. The whey cheese samples were mixed (1:10, *w*/*v*) with buffered peptone water (Liofilchem, Teramo, Italy) and submitted to 10 decimal serial dilutions. The LAB counts were enumerated on M17 agar (Oxoid, Basingstoke, Hampshire, UK) and incubated at 30 °C for 72 h [38]. The yeasts and molds were enumerated on potato dextrose agar (PDA) (Oxoid, UK), which was acidified with sterile lactic acid (pH 4.5) (Lab M Ltd., Heywood, UK) to inhibit bacteria growth after incubation at 25 °C for 120 h, followed by microscopic confirmation tests for each type of colony encountered [39]. The coliforms were enumerated on violet red bile agar (Liofilchem, Teramo, Italy) at 30 °C for 24 h [40]. The Enterobacteria were enumerated on violet red bile glucose agar (Liofilchem, Teramo, Italy) at 37 °C for 24 h [41].

### 2.5. Physicochemical Analysis

The cheeses were analyzed in triplicate at 1, 7, and 14 days of storage. The following physicochemical properties were determined according to prescribed methods: dry matter and moisture [42], ash [43], fat [44], protein [45], fiber [46], and lactic acid [47]. The content of sugars (saccharose, glucose, fructose, lactose, and total sugars) was determined with high-performance liquid chromatography (HPLC), as previously described in detail by Mileriene et al. [48].

The pH was measured using a portable pH meter (Sartorius Professional meter for pH Measurement, Germany) by inserting the electrode directly into the cheese sample. Three readings were taken for each triplicate at room temperature.

The texture of the whey cheese was assessed using Texture analyzer CT3 (Brookfield, WI, USA) [49]. Cylindrical plastic containers (60.0 mm diameter) were filled with the acid whey cheese up to 20 mm height for testing. For each measurement, the acid whey cheese sample was penetrated in its center with a cylinder probe (38.1 mm diameter) to a depth of 10 mm (50% deform), using a probe SPEED of 1 mm/s. Each sample was evaluated in triplicate.

The color changes were measured using a CIE L* a* b* color system using a portable chroma meter CR-400 (Konica Minolta, Tokyo, Japan). L* is a measure of lightness, a* is a measure of redness (or—a* of greenness), and b* is a measure of yellowness (or—b* of blueness). A standard white plate was used to calibrate the equipment, with color coordinates L_standard_ = 97.6, a_standard_ = 0.01, and b_standard_ = 1.60. Three readings were taken for each triplicate. The overall color change (ΔE) was calculated as described by Mileriene et al. [21] as follows:

∆E = [(L − L_0_)2 + (a − a_0_)2 + (b − b_0_)2]^1/2^

where L_0_, a_0_, and b_0_ were the values of day 1, and L, a, and b were the values measured throughout the storage period.

The hue angle (h*), which refers to the degree of the dominant spectral component (red, green, and blue) and ranges from 0° to 360°, and the chroma (C*), which represents the saturation of a color, were calculated according to Guiné et al. [50] as follows:

h* = arctg (b*/a*), for a* > 0; b* > 0;

h* = 180 + arctg (b*/a*), for a* < 0; b* > 0;

C* = √ (a*2 + b*2)

### 2.6. Assessment of Proteolysis

Cheese proteolysis was evaluated by measuring pH 4.6-soluble nitrogen, which was expressed as a percentage of the total nitrogen (pH 4.6-SN/TN), according to Fenelon et al. [51]. The pH 4.6-soluble fractions of the cheese samples were prepared to modify the procedure of Kuchroo and Fox [52], as described by Sousa and McSweeney [53]. The quantity of the total free amino acids in the pH 4.6-soluble fractions of the cheese was assessed using trinitrobenzene sulfonic acid (TNBS). The separate free amino acids in the cheese samples after 1 and 14 days of storage were evaluated using an amino acid analyzer, according to Fenelon et al. [51]. They were extracted with dilute hydrogen chloride acid. The co-extracted nitrogen-containing macromolecules were precipitated with sulfosalicylic acid and separated by filtration. The pH of the filtered solution was determined to be 2.20. The amino acids were separated by ion exchange chromatography and determined photometrically by a reaction with ninhydrin at a wavelength of 570 nm (440 nm for proline). The changes in mean concentrations of the free amino acids in the cheese samples were calculated in Microsoft Excel (2016), and then a heatmap was generated using online tool Heatmapper (http://www.heatmapper.ca/expression/, accessed on 1 February 2023. The scale-type parameter was selected as “none”) to visualize the obtained results. The tools of the obtained results were the changes during the 14-day storage of the acid whey cheese.

### 2.7. Determination of Volatile Fatty Acids

The amount of volatile fatty acids was determined at 1 and 14 days of cheese storage by headspace solid-phase microextraction (HS-SPME) gas chromatography–mass spectrometry (GC–MS) analysis, as described in detail by Mileriene et al. [48] using a GC–MS (6890 N GC, 5973 NetworkedMS MSD, Agilent Technologies, Santa Clara, CA, USA) equipped with an HP-5MS column (30 m, 0.25 mm i.d., 0.25 µm film thickness). Semi-quantification of volatile compounds was based on 4-methyl-2-pentanol (Sigma-Aldrich, Schnelldorf, Germany), which was used as an internal standard.

### 2.8. Sensory Evaluation

The overall sensory acceptability of the whey cheese samples (20 g) was evaluated by a trained panel [54] of 10 individuals (aged 30–55) at the Sensory Laboratory of the Lithuanian University of Health Sciences. The same group of panelists were present during both of the sensory analysis sessions at storage days 1 and 14. The cheese samples were rated using a 10-point scale ranging from 1 (poor) to 10 (excellent).

### 2.9. Statistical Analysis

The data analysis was performed with SPSS (24.0, Chicago, IL, USA, SPSS Inc.) software. Every treatment was run in triplicate. Significant differences among the means were determined by two-way analysis of variance (ANOVA) and the Tukey test (*p* < 0.05).

## 3. Results and Discussion

### 3.1. Composition of Materials and Cheese Samples

Before the sample preparation, the composition of the separate materials was determined. The freeze-dried and powdered apple pomace contained 91.8% dry matter, 3.9% fat, 3.1% protein, 14.9% saccharose, 6.7% glucose, 23.3% fructose, 45.9% total sugars, 30.5% dietary fiber, and 83.5% total carbohydrates. The composition of the produced acid whey cheese as a control sample (C) is shown in Table 1.

The subsequent composition of the acid whey cheese with probiotic, prebiotic, and synbiotic supplementation during the 14 days of storage is presented in Table 1. The storage day and the sample factors had a significant impact on all of the cheese components, except ash (D*S effect was not significant), and a strong interaction was also observed. On day one, the freeze-dried apple pomace significantly affected the moisture content. In general, the supplementation of the cheese with apple pomace (containing 91.8% dry matter) resulted in reduced moisture content (cheese C + AP) (68.64 ± 0.13%) and reduced protein density (16.98 ± 0.0%,) compared to the non-supplemented control samples (71.20 ± 0.0%, 17.49 ± 0.01%, respectively). This change in moisture content also affected the other parameters. The moisture content increased, while the protein content decreased in the cheese with *L. lactis* LL16 (C + LL16) compared to the control. The addition of *L. lactis* LL16 strain increased the moisture content in the pomace-supplemented cheese (C + AP 68.64 ± 0.13; C + AP + LL16 70.58 ± 0.05, day one) as well, thus balancing out the drying effect of the apple pomace powder. Significant decreases in protein content on the last day of storage were observed only in the *L. lactis* LL16- (C + LL16-) supplemented samples.

### 3.2. Assessment of Proteolysis

The storage day, the cheese sample, and their interaction had a significant effect on all of the proteolytic parameters in our study (*p* < 0.001). A higher proteolysis rate, which is expressed as concentrations of pH 4.6-soluble nitrogen (pH 4.6-SN) and of free amino acids (FAA, Table 2), was noted in the control and the AP + LL16-supplemented cheeses on day one, and then decreased at the end of storage (*p* < 0.001), compared to the opposite trend in cheeses C + AP and C + LL16. As expected, no considerable proteolysis was noticed in the control acid whey cheese during the 14 days of storage, as also reported in other studies [29,55], since Ricotta-type whey cheeses have high levels of whey proteins, which are resistant to hydrolysis by plasmin [56]. However, proteolytic activity was observed in the C + AP sample. This can be explained by the results of Koak et al. [57], where the proteases that are naturally present in apples showed minimal proteolytic activity of casein. The considerable variation in the extent and nature of proteolysis among the supplemented acid whey cheese samples during storage is thought to be attributable to the differences in the speed and the degree of degradation of proteins by the action of probiotics and synbiotics [58]. The addition of the presumptive probiotic *L. lactis* LL16 strain to the plain acid whey cheese slightly increased the proteolysis throughout the storage period. The highest concentration of FAA was detected in the samples that were supplemented with AP and *L. lactis* LL16 strain after one day of storage, which decreased significantly at the end of the storage period. Previous studies have shown that the amino acids that are released from peptides by the action of peptidases subsequently may act as precursors for catabolic reactions, which produce many important volatile flavor compounds [59,60].

**Figure 2:**
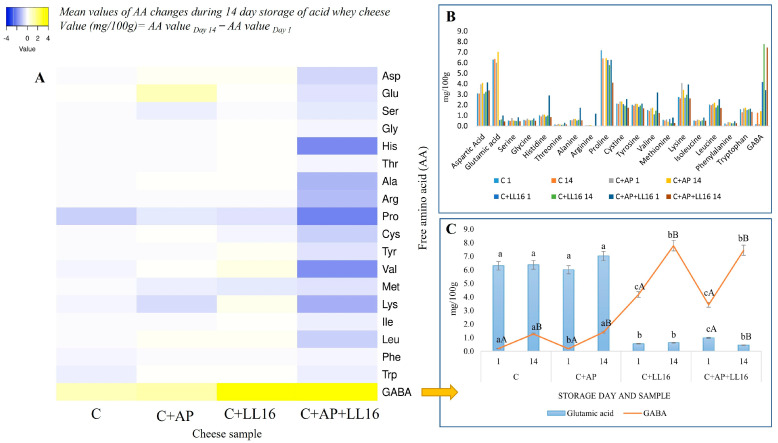
Free amino acid (AA) profile of acid whey cheese samples (C, control acid whey cheese; C + AP, cheese made with 3% of apple pomace AP; C + LL16, cheese made with 0.2% *L. lactis* LL16 strain biomass (100 g/0.2 g; C + LL16); C + AP + LL16, cheese made with 3% of apple pomace and 0.2% *L. lactis* LL16, during 14 days of storage at 4 °C). (**A**) Heatmap of AA changes. (**B**) Mean values of AA. (**C**) Mean values of glutamic acid and GABA. The means with different uppercase letters (A,B) indicate significant differences (*p* < 0.05) between the storage days for each treatment. The means with different lowercase letters (a–c) indicate significant differences (*p* < 0.05) between the treatments at the same time interval (day).

The concentrations of aspartic and glutamic acid, leucine, cysteine, tyrosine, lysine, and proline were higher than those of the other amino acids in the control and the AP-supplemented samples (Figure 2B), while threonine, arginine, phenylalanine, and isoleucine were present at lower concentrations compared to the other amino acids throughout the storage period. A similar trend has been observed by other authors [56]. The supplementation of acid whey cheese with *L. lactis* LL16 had a distinct impact on the free amino acids profile (Figure 2).

There was six-fold less glutamic acid in the *L. lactis* LL16-supplemented samples (*p* < 0.001) compared to the non-supplemented samples; while the content of GABA significantly (*p* < 0.001) increased in the *L. lactis* LL16-supplemented samples, especially, after 14 days of storage, reaching an average of 7.61 mg/100 g cheese (Figure 2C).

It has been reported that *L. lactis* has a complex proteolytic system [61], with some strains having the ability to convert glutamic acid to bioactive GABA [24,62], which is a non-protein amino acid that has been shown to have an effect on brain function by preventing or reducing anxiety, depression, insomnia, and memory loss; moreover, it stimulates the immune system and prevents inflammatory processes, hypertension, and diabetes [23]. The conversion of glutamate to GABA is catalyzed by glutamate decarboxylase (GAD) and the reaction requires pyridoxal-5′-phosphate (PLP) as a cofactor. A wide range of LAB have the potential to produce GAD [63].

Santos-Espinosa et al. [24] found that 37% of the *Lactococcus* spp. strains that were isolated from Mexican cheeses showed GAD enzymatic activity in the fermented milk. *Lactococcus* spp. strain L-598 showed a GABA production of 11.0 mg/L and was not significantly different (*p* > 0.05) from those of *Lactobacillus* spp. that were assessed. While *Lactococcus* spp. strains L-571 and L-572 produced the highest concentration of GABA (86.0 and 86.2 mg/L, respectively), and were significantly different (*p* < 0.05) from all of the other fermented milk samples [24].

The most vigorous release of free AA was observed in the synbiotic cheese C + AP + LL16, which presented with double- and triple-fold amounts of histidine, valine, arginine, leucine, lysine, and alanine, compared to those in other cheeses (Figure 2A). We speculate that the most intense decline of AA in the C + AP + LL16 sample was influenced by the combined proteolytic effect of the proteases that are present in AP and the proteolytic activity of *L. lactis* LL16. Further analysis is needed in order to evaluate these aspects in more depth.

### 3.3. Sugar Profile

The content of sugars (saccharose, glucose, fructose, and lactose), fiber, lactic acid, and the pH (Table 3) were determined in the acid whey cheese samples during storage. The storage day, the sample treatment, and their interaction significantly affected the contents of carbohydrates. The addition of apple pomace fortified the whey cheese samples with sucrose, glucose, fructose, and fiber. The addition of *L. lactis* LL16 significantly decreased the amounts of lactose, glucose, and total sugars, however, had no impact on the amounts of saccharose, fructose, or fiber.

Interestingly, we observed a significant degradation of lactic acid contents in all of the cheese samples. The obtained results are intriguing, because usually the dynamics of lactic acid content in cheese are directly caused by the presence of microorganisms. In dairy products, LAB are usually responsible for the increase in lactic acid, due to the fermentation of lactose and other simple sugars, while the decrease in lactic acid is more likely linked to the growth of contaminant microflora, such as yeasts and molds. However, since the contaminant microflora was below detectable levels in our whey cheese samples (see Section 3.4), we speculate that the degradation of lactic acid might have been caused by the activity of the thermostable enzymes that are naturally present in whey cheese and apple pomace. Enzymes, such as lactases, are produced during milk fermentation. It is well known that acid whey, as a by-product of heat-treated fermented milk, contains a high amount of enzymes, such as lactase [64]. We speculate, that these enzymes remained active during the production of the acid whey cheese and its pasteurization, since other studies [65] claim that the presence of sugars and proteins can significantly protect the enzyme lactase from inactivation at extreme thermal and pH conditions.

During the 14 days of storage, the pH remained stable in all of the samples, except C + LL16, where a significant increase at day seven, and a decrease at day 14, were observed. Similarly, Borba et al. [66] reported that the pH values of creamy ricotta increased between one and seven days of storage, but remained stable until 14 days of storage. A rapid post-production reduction in the lactate–protein ratio and the concentration of calcium phosphate can result in a pH rise during storage [67]. The significant increase in pH in the C + LL16 sample also might have been caused by the proteolytic activity of *L. lactis* LL16, due to the produced amino acids.

Therefore, further research is needed in order to evaluate enzymatic residues and their interaction with the fermentation activity of the probiotics that are used in sustainable acid whey cheese production.

### 3.4. Microbiological Analysis

For dairy products, microorganisms have a particularly important role and are significant for the physicochemical and sensory characteristics and the shelf life of the final product [68]. M17 medium was used to estimate the growth of *L. lactis* LL16 in the pasteurized control and the experimental cheeses (Table 4). The results show that the desired effect of pasteurization was obtained, since the samples without added *L. lactis* LL16 (C and C + AP) had LAB counts that were below the detectable level of 1 log cfu/mL. Moreover, the undesirable microorganisms (Enterobacteriaceae, total coliforms, and fungi) were also below the detectable limit in all of the samples (data not shown). The presumptive probiotic strain *L. lactis* LL16 maintained mean counts of over 6 log cfu/g for seven days. Our study showed that the storage day, the cheese sample, and their interaction significantly affected the LAB growth. A gradual decrease in LAB counts was observed during refrigerated storage in both the C + LL16 and the C + AP + LL16 samples. Slightly, though significantly, higher counts of LAB we detected in the apple pomace-supplemented samples on day seven (*p* < 0.05), suggesting a positive impact of apple pomace components on strain survival. No statistical difference was observed between these two cheese treatments at the end of storage (on day 14). A study by Beermann et al. [69] showed that the polyphenols that are present in apple pomace extract can have a concentration-dependent manner both to enhance and inhibit the growth of several LAB.

### 3.5. Volatile Fatty Acid Content

The volatile compounds that are responsible for the typical aroma of cheese are produced mainly by lipolytic and proteolytic pathways and by the metabolism of lactose, lactate, and citrate [70]. Secondary biochemical mechanisms are involved in the metabolism of fatty acids and amino acids, which directly contribute to the synthesis of many volatile fatty acids and influence cheese flavor [71].

Acetic, propionic, and butyric acids were the volatile fatty acids (VFA) that were detected in the whey cheese samples (Table 5). The amount of acetic acid was significantly higher among all of the identified VFA. Li et al. [72] found that cheese samples that were fermented with different LAB (*Streptococcus thermophilus* B8, *Lactobacillus helveticus* B6, *Weissella confusa* B14, and *Lactobacillus rhamnosus* B10) contained the highest concentration of acetic acid between all 12 of the identified VFA as well [72].

The storage day, the cheese sample, and their interaction significantly affected the VFA content (*p* < 0.001). A higher (*p* < 0.05) amount of acetic acid was found in the control cheese compared to the AP-supplemented cheeses (C + AP and C + AP + LL16) on day one. This may be related to the lower amount of cheese matrix in the AP-supplemented samples, suggesting that the content of acetic acid in the whey cheese matrix is naturally higher than that of AP. The VFA content that was found in the control sample could be predisposed to previous lactose fermentation by the starter LAB during sour curd production. The primary conversion of lactose leads to the formation of lactic acid. Still, a fraction of the intermediate pyruvate can be converted to various flavor compounds, such as acetic acid [73].

The acid whey cheese with *L. lactis* LL16 (C + LL16) occupied an intermediate position between the samples according to the acetic acid content and did not differ significantly from the remaining samples. On day 14, the content of acetic acid decreased (*p* < 0.05) in the C, the C + AP, and the C + LL16 samples, while it significantly increased (*p* < 0.05) in the symbiotic cheese that was supplemented with AP and LL16. This result suggests the positive impact of apple pomace components on LL16 strain survival. The content of propionic and butyric acid was significantly lower in all of the cheese samples compared to the acetic acid and did not differ in cheeses between the storage days.

### 3.6. Texture and Color Assessment

The texture and the color of the acid whey cheese with probiotic, prebiotic, and synbiotic supplementation are presented in Table 6. The texture was significantly influenced by the storage day, the cheese sample, and their interaction (*p* ≤ 0.01). The significant gradual decrease in hardness during storage was observed only in the apple pomace-supplemented sample. The softening effect might be attributed to the higher ratio of moisture to protein and to the increase in filler volume that results in a decrease in the amount of protein matrix [74]. An increase in hardness was detected only in the synbiotic-supplemented cheese (C + AP + LL16), while it decreased on day 7 and increased on day 14 in control cheese C. On the contrary, the hardness of the *L. lactis* LL16-supplemented cheese increased on day 7 and decreased on day 14. These trends seem to result from a complex interaction of several variables. The interactions between apple pomace and cheese matrix need further investigation.

Table 6 shows the values of the color coordinates (L*, a*, b*, and ΔE). All of the cheese samples presented an average L* value of 91.85 on day one, which was very close to that reported by Ortiz et al. [75] for full fat (16.4%) and reduced fat (1.6%) ricotta cheese, which were 89.00 ± 2.00 and 90.00 ± 1.00, respectively. Samples C and C + LL16 did not differ significantly (except on day one) from each other but were significantly (*p* ≤ 0.05) brighter (avg. 101.65 versus 94.95) than the apple pomace-supplemented samples C + AP and C + AP + LL16 during the entire study. Thus, apple pomace lowered the lightness of the supplemented cheeses samples. A 2022 study found that, as the apple pomace concentration increased (from 0.2% to 1.0%), the lightness (L*) values decreased from 77.27 to 73.36 in yogurt samples [76].

However, during the refrigerated 14-day storage, the L* value increased significantly in all of the acid whey cheese samples, compared to day one. Ortiz et al. [75] did not reveal such a significant change in the L* value during the storage of ricotta.

Supplementing cheese with LAB can impact its color during storage [77]. The acid whey cheese that was supplemented with *L. lactis* LL16 was significantly brighter (96.49 ± 1.30) than all of the remaining samples (avg. 90.30) on day 1; however, it did not differ significantly from the control on the subsequent days of the study. Lower values for brightness (L*) and higher values for green color (a*) were found in the probiotic-supplemented (*L. acidophilus* La-05 and *B. lactis* Bb-12) goat ricotta samples in Meira et al. [78]. The authors state that the difference between the samples might be associated with the ability of some LAB strains to synthesize the B vitamins that impact the product’s green color [79]. This study revealed a higher value of a* in the control cheese than in the probiotic-supplemented C + LL16 sample, but only on day 1; later, the mentioned samples did not differ significantly during the storage period. Overall, samples C and C + LL16 had a negative value of a*, indicating the presence of a greenish coloration instead of reddish. Both of the apple pomace-supplemented samples (C + AP and C + AP + LL16) were significantly more reddish than the unsupplemented samples; still, the red color of sample C + AP was more intense (*p* ≤ 0.05) than that of C + AP + LL16 (avg. 2.21 versus 1.62), especially when in storage for 7–14 days. Popescu et al. [76] found that yogurt supplementation with apple pomace significantly reduced the greenish coloration in samples.

**Figure 3:**
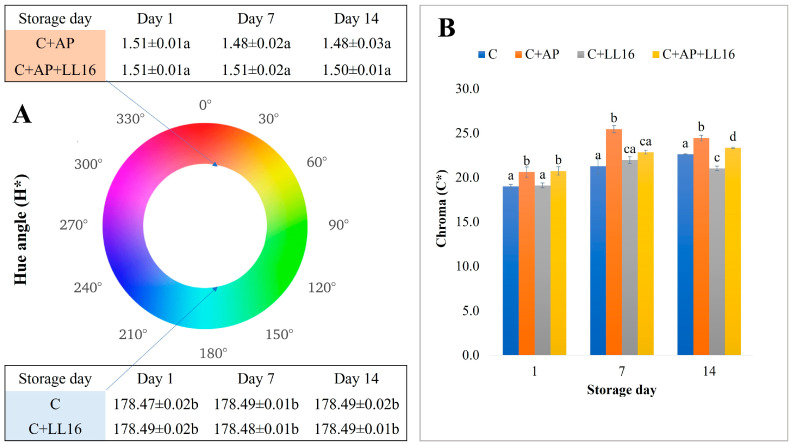
Color coordinates hue angle (**A**) and chroma (**B**) evaluated in the acid whey protein cheese samples during 14 days of storage at 4 °C. C, control acid whey cheese; C + AP, cheese made with 3% of apple pomace AP; C + LL16, cheese made with 0.2% *L. lactis* LL16 strain biomass (100 g/0.2 g; C + LL16); C + AP + LL16, cheese made with 3% of apple pomace and 0.2% *L. lactis* LL16. The means with different lowercase letters (a–c) indicate significant differences (*p* < 0.05) between the treatments at the same time interval (day).

The results that were obtained for the a* values complied with the hue angle (h*) estimated values (Figure 3A) showed the distribution of cheeses into green and red zones, depending on the sample type.

According to Guiné et al. [50], for values of a hue angle that are near 0° or 360°, the tone is red, while for a hue angle of around 180°, the tone is green. Thus, the apple pomace-supplemented cheeses C + AP and C + AP + LL16 entered the red zone, while the unsupplemented cheeses (C and C + LL16) entered the green area extremely clearly in this study.

All of the acid whey cheese samples’ values of the coordinate b* were positive, suggesting the predominance of a yellow color instead of blue. Similar values of b* were found by Guiné et al. [50], where the color of novel fresh cheese with raspberry, blueberry, or their mix was evaluated. However, the samples with apple pomace (C + AP and C + AP + LL16) were significantly yellower compared to C and C + LL16 in this study. The same pattern of b* coordinate was found in a study by Popescu et al. [76], in which the yellow color was more intense when a higher apple pomace concentration was added to yogurt samples.

Figure 3B represents the values of chroma for all of the samples that were evaluated. Based on the results of Guiné et al. [50], higher chroma values indicate higher purity of color, while the lowest values (close to zero) correspond to more pale colors.

According to the chroma (C*) value, the acid whey cheese samples that were supplemented with apple pomace (C + AP and C + AP + LL16) represented a higher purity of color throughout the study. By the end of the storage period, the control sample expressed significantly higher (*p* ≤ 0.05) overall color change (ΔE) values, compared to the rest of the samples (Table 6).

### 3.7. Overall Sensory Acceptability

**Figure 4:**
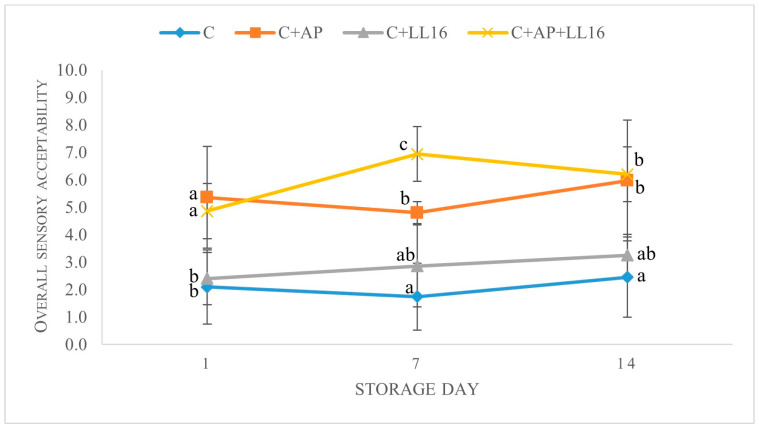
Overall sensory acceptability of the acid whey cheese samples during 14 days of storage at 4 °C. C, control acid whey cheese; C + AP, cheese made with 3% of apple pomace AP; C + LL16, cheese made with 0.2% *L. lactis* LL16 strain biomass (100 g/0.2 g; C + LL16); C + AP + LL16, cheese made with 3% of apple pomace and 0.2% *L. lactis* LL16. The means with different lowercase letters (a–c) indicate significant differences (*p* < 0.05) between the treatments at the same time interval (day).

Studies suggest that supplementation of cheese with probiotic-type strains [80] and prebiotics [81] may positively affect the sensory characteristics. The overall sensory acceptability is presented in Figure 4. As expected, the cheese that was produced with apple pomace (C + AP) was particularly appreciated by the judges, as highlighted by the highest acceptability scores throughout the storage period, compared to both of the unsupplemented samples (*p* < 0.05). The addition of *L. lactis* LL16 to the AP-supplemented cheese (C + AP + LL16) demonstrated a stable increase in acceptability from day one to day seven (*p* < 0.05). These samples were described as “fresh” and “fruity”. According to Madrera et al. [82], 132 volatile compounds were generated during the fermentation of apple pomace with *S. cerevisiae, H. valbyensis, H. uvarum,* and a combination of *S. cerevisiae* and an inoculated enzyme, indicating that fermented apple pomace can be used as a natural flavoring. This is in agreement with our findings, demonstrating a significant increase in volatile acetic acid content in the synbiotic samples (Table 5).

We also detected a slight, though insignificant, increase in the overall acceptability in plain curd cheese carrying *L. lactis* LL16 (*p* ≥ 0.05). The supplementation of the curd with *L. lactis* LL16 had no significant impact on the rate of cheese proteolysis (Table 2); however, other studies show that *L. lactis* has a complex proteolytic system that, together with other proteolytic enzymes, can convert casein into peptides and amino acids, which are the key precursors of volatile flavor compounds, influencing cheese sensory properties [61].

## 4. Conclusions

The development of novel food and/or functional food products with presumptive probiotic microorganisms, soluble dietary fibers, or both, is increasingly challenging, as it must fulfill the consumer’s expectations for products that are simultaneously palatable and health-promoting. The applications that have been described in this study have shown the high potential of valorizing acid-curd cheese and apple juice industry by-products for the development of sustainable, innovative, and beneficial dairy desserts as a potential probiotic carrier to fulfill market niches. The presumptive probiotic strain *L. lactis* LL16 that was used in this study was able to maintain mean counts of over 6 log CFU/g during the cold storage of the acid whey cheese. The addition of apple pomace, containing 46% of the easily assimilable carbohydrates, contributed to the survival of the strain, significantly enhanced the sensory perception of the product, and replaced the sugar or the other taste and texture enhancers that are commonly used in this type of dairy dessert. The utilization of the agro-industrial waste, apple pomace powder, as a texture and flavor enhancer in curd desserts enables us to lock up to 30% of leftover acid whey into the final product, thus contributing to the circular economy as well. Additionally, the consumption of cheese that is enriched with 3% apple pomace (containing 30% fiber) leads to an increased daily fiber intake. As the enrichment of acid whey cheese with *L. lactis* LL16 increased the amount of γ-aminobutyric acid (GABA) significantly, functional acid whey cheese may be a healthy food option, as probiotic microorganisms are known to positively affect brain function, stimulate immune response, and prevent other diseases, such as hypertension and diabetes. However, as color changes and an increase in hardness were detected in the synbiotic acid whey cheese, additional technological operations, such as homogenization, are required in order to further improve the sensory quality characteristics.

## Figures and Tables

**Table 1:** Composition of the acid whey cheese samples during 14 days of storage at 4 °C (values reported in g/100 g).

Parameter	Storage Time (Days)	Cheese Treatments			
		C	C + AP	C + LL16	C + AP + LL16
Moisture	1	71.20 ± 0.00 aA	68.64 ± 0.13 b	71.72 ± 0.00 cA	70.58 ± 0.05 dA
	7	70.30 ± 0.04 aB	68.92 ± 0.16 b	72.41 ± 0.03 cB	69.86 ± 0.14 dB
	14	71.15 ± 0.04 aA	69.07 ± 0.03 b	72.55 ± 0.02 cB	70.27 ± 0.05 dA
Protein	1	17.49 ± 0.01 aA	16.98 ± 0.00 bA	17.33 ± 0.01 cA	16.32 ± 0.01 dA
	7	18.30 ± 0.01 aB	17.92 ± 0.00 bB	17.48 ± 0.01 cB	17.27 ± 0.02 dB
	14	18.29 ± 0.01 aB	17.71 ± 0.00 bB	17.19 ± 0.01 cC	17.63 ± 0.01 dC
Fat	1	5.18 ± 0.03 aA	5.48 ± 0.04 b	5.30 ± 0.01 ab	5.15 ± 0.10 a
	7	5.62 ± 0.07 aB	5.51 ± 0.01 a	5.32 ± 0.04 b	5.25 ± 0.03 c
	14	5.62 ± 0.00 aB	5.49 ± 0.03 b	5.44 ± 0.01 b	5.31 ± 0.01 c
Ash	1	0.72 ± 0.04	0.76 ± 0.01	0.71 ± 0.01	0.71 ± 0.01
	7	0.72 ± 0.00	0.73 ± 0.00	0.71 ± 0.00	0.72 ± 0.00 A
	14	0.71 ± 0.00	0.71 ± 0.00	0.70 ± 0.00	0.69 ± 0.00 B

The results are the means of three replicates ± standard error, and the following abbreviations are used: C, control acid whey cheese; C + AP, cheese made with 3% of apple pomace AP; C + LL16, cheese made with 0.2% *L. lactis* LL16 strain biomass (100 g/0.2 g; C + LL16); C + AP + LL16, cheese made with 3% of apple pomace and 0.2% *L. lactis* LL16. The means with different capital letters (A–C) indicate significant differences (*p* < 0.05) among the storage days for each treatment. The means with different lowercase letters (a–d) indicate significant differences (*p* < 0.05) between the treatments at the same time interval (day).

**Table 2:** Mean values for proteolysis parameters of the acid whey cheese samples during 14 days of storage at 4 °C.

Variables	Storage Time (Days)	Cheese Treatments			
		C	C + AP	C + LL16	C + AP + LL16
pH 4.6-soluble Nitrogen (g/100 g)	1	1.20 ± 0.01 * a	1.09 ± 0.00 * b	0.91 ± 0.00 * c	1.45 ± 0.00 * d
	14	1.08 ± 0.01 * a	1.28 ± 0.00 * b	0.94 ± 0.00 * c	0.79 ± 0.00 * d
Free Amino Acids (mg/100 g)	1	3.29 ± 0.01 * a	2.91 ± 0.00 * b	2.48 ± 0.00 * c	3.71 ± 0.00 * d
	14	3.09 ± 0.00 * a	3.56 ± 0.00 * b	2.54 ± 0.00 * c	2.18 ± 0.00 * d

The results are the means of three replicates ± standard deviation, and the following abbreviations are used: C, control acid whey cheese; C + AP, cheese made with 3% of apple pomace AP; C + LL16, cheese made with 0.2% *L. lactis* LL16 strain biomass (100 g/0.2 g; C + LL16); C + AP + LL16, cheese made with 3% of apple pomace and 0.2% *L. lactis* LL16. The means with * indicate significant differences (*p* < 0.05) between the storage days for each treatment. The means with different lowercase letters (a–d) indicate significant differences (*p* < 0.05) between the treatments at the same time interval (day).

**Table 3:** Evolution of sugars, fiber, pH, and lactic acid in the acid whey cheese samples during 14 days of storage at 4 °C.

Parameter	Storage Time (Days)	Samples			
		C	C + AP	C + LL16	C + AP + LL16
Saccharose, %	1	nd *	0.61 ± 0.01	nd	0.59 ± 0.05
	7	nd	0.56 ± 0.01	nd	0.59 ± 0.00
	14	nd	0.61 ± 0.03 a	nd	0.52 ± 0.03 b
Glucose, %	1	nd	0.34 ± 0.02 aA	nd	0.24 ± 0.02 bA
	7	nd	0.15 ± 0.00 B	nd	0.14 ± 0.04 B
	14	nd	0.11 ± 0.02 B	nd	0.12 ± 0.03 B
Fructose, %	1	nd	0.79 ± 0.01 a	nd	0.75 ± 0.01 b
	7	nd	0.75 ± 0.03 a	nd	0.67 ± 0.02 b
	14	nd	0.73 ± 0.03	nd	0.65 ± 0.04
Lactose, %	1	3.57 ± 0.02	3.63 ± 0.01	3.64 ± 0.04 A	3.57 ± 0.02 A
	7	3.52 ± 0.01 a	3.55 ± 0.03 a	3.09 ± 0.02 bB	3.21 ± 0.02 aB
	14	3.54 ± 0.02	3.57 ± 0.02	3.16 ± 0.06 B	3.10 ± 0.05 B
Total sugars, %	1	3.57 ± 0.02 a	5.37 ± 0.01 b	3.64 ± 0.04 aA	5.13 ± 0.01 bA
	7	3.22 ± 0.01 a	5.27 ± 0.01 b	3.20 ± 0.00 aB	4.60 ± 0.04 bB
	14	3.27 ± 0.03 a	5.20 ± 0.01 b	3.16 ± 0.06 aB	4.39 ± 0.15 bB
Fiber, %	1	nd	1.10 ± 0.00	nd	1.15 ± 0.07
	7	nd	1.20 ± 0.00	nd	1.15 ± 0.07
	14	nd	1.20 ± 0.00	nd	1.20 ± 0.00
pH	1	4.68 ± 0.01 a	4.59 ± 0.02 b	4.69 ± 0.01 aA	4.72 ± 0.05 a
	7	4.66 ± 0.03 a	4.66 ± 0.01 a	4.78 ± 0.02 bB	4.72 ± 0.02
	14	4.69 ± 0.01	4.62 ± 0.07	4.71 ± 0.03 A	4.66 ± 0.04
Lactic acid, mg/100 g	1	804.50 ± 2.12 aA	703.50 ± 3.54 bA	718.50 ± 0.71 cA	719.00 ± 1.41 dA
	7	790.00 ± 2.83 aB	761.00 ± 2.83 bB	706.50 ± 3.54 cA	666.50 ± 2.12 dB
	14	584.50 ± 2.12 aC	681.00 ± 11.31 bA	568.50 ± 6.36 aB	680.50 ± 10.61 bB

The results are the means of three replicates ± standard deviation, and the following abbreviations are used: C, control acid whey cheese; C + AP, cheese made with 3% of apple pomace AP; C + LL16, cheese made with 0.2% *L. lactis* LL16 strain biomass (100 g/0.2 g; C + LL16); C + AP + LL16, cheese made with 3% of apple pomace and 0.2% *L. lactis* LL16. The means with different uppercase letters (A–C) indicate significant differences (*p* < 0.05) between the storage days for each treatment. The means with different lowercase letters (a–d) indicate significant differences (*p* < 0.05) between the treatments at the same time interval (day). nd *—not detected/below detection limit.

**Table 4:** Lactic acid bacteria count (log cfu/mL) of the acid whey cheese samples during 14 days of storage at 4 °C.

Media	Storage Time (Days)	Samples			
		C	C + AP	C + LL16	C + AP + LL16
M17	1	<1	<1	6.66 ± 0.02 A	6.65 ± 0.17 A
	7	<1	<1	6.18 ± 0.08 * B	6.32 ± 0.02 * A
	14	<1	<1	5.28 ± 0.26 C	5.51 ± 0.43 B

The results are the means of three replicates ± standard deviation, and the following abbreviations are used: C, control acid whey cheese; C + AP, cheese made with 3% of apple pomace AP; C + LL16, cheese made with 0.2% *L. lactis* LL16 strain biomass (100 g/0.2 g; C + LL16); C + AP + LL16, cheese made with 3% of apple pomace and 0.2% *L. lactis* LL16. The means with different capital letters indicate significant differences (*p* < 0.05) among the storage days for each treatment. The means with * (*p* < 0.05) indicate a significant difference between the treatments at the same time interval (day).

**Table 5:** Content of volatile fatty acids of the acid whey cheese samples during 14 days of storage at 4 °C.

Volatile Fatty Acids, ppm	Storage Time (Days)	Samples			
		C	C + AP	C + LL16	C + AP + LL16
Acetic acid	1	3.06 ± 0.01 aA	2.97 ± 0.01 bA	3.04 ± 0.02 abA	2.98 ± 0.01 bA
	14	2.60 ± 0.03 aB	2.70 ± 0.01 bB	2.76 ± 0.03 bB	3.04 ± 0.01 cB
Propionic acid	1	0.04 ± 0.00	0.04 ± 0.01	0.04 ± 0.00	0.03 ± 0.01
	14	0.03 ± 0.00	0.04 ± 0.01	0.03 ± 0.00	0.03 ± 0.00
Butyric acid	1	0.13 ± 0.00	0.12 ± 0.01	0.14 ± 0.01	0.13 ± 0.01
	14	0.11 ± 0.00 a	0.13 ± 0.00 b	0.12 ± 0.00 ab	0.11 ± 0.01 ac

The results are the means of three replicates ± standard deviation, and the following abbreviations are used: C, control acid whey cheese; C + AP, cheese made with 3% of apple pomace AP; C + LL16, cheese made with 0.2% *L. lactis* LL16 strain biomass (100 g/0.2 g; C + LL16); C + AP + LL16, cheese made with 3% of apple pomace and 0.2% *L. lactis* LL16. The means with different uppercase letters (A–B) indicate significant differences (*p* < 0.05) between the storage days for each treatment. The means with different lowercase letters (a–c) indicate significant differences (*p* < 0.05) between the treatments at the same time interval (day).

**Table 6:** Texture and color parameters of the acid whey protein cheese samples during 14 days of storage at 4 °C.

Parameters	Storage Time (Days)	Cheese Treatments			
		C	C + AP	C + LL16	C + AP + LL16
Texture, mJ	1	0.33 ± 0.06 aA	1.03 ± 0.06 bA	0.40 ± 0.00 aA	0.40 ± 0.01 aA
	7	0.20 ± 0.00 aB	0.80 ± 0.20 bAB	0.70 ± 0.10 bB	0.40 ± 0.00 aA
	14	0.47 ± 0.06 aC	0.63 ± 0.06 bB	0.57 ± 0.06 aC	0.70 ± 0.00 bB
L*	1	91.98 ± 0.69 aA	89.08 ± 2.70 aA	96.49 ± 1.30 bA	89.85 ± 1.67 aA
	7	103.99 ± 5.89 aB	97.11 ± 1.57 bB	107.79 ± 1.78 aB	98.56 ± 0.79 bB
	14	105.30 ± 1.15 aB	96.46 ± 0.28 bB	104.39 ± 0.91 aB	98.66 ± 0.62 cB
a*	1	−0.84 ± 0.09 aA	1.31 ± 0.07 bA	−1.18 ± 0.11 c	1.27 ± 0.03 bA
	7	−1.40 ± 0.16 aB	2.24 ± 0.10 bB	−1.15 ± 0.05 a	1.47 ± 0.07 cB
	14	−1.29 ± 0.06 aB	2.17 ± 0.11 bB	−1.29 ± 0.11 a	1.76 ± 0.11 cC
b*	1	18.97 ± 0.25 aA	20.58 ± 0.57 bA	19.07 ± 0.27 aA	20.70 ± 0.47 bA
	7	21.24 ± 0.92 aB	25.35 ± 0.41 bB	21.95 ± 0.39 aB	22.80 ± 0.21 cB
	14	22.58 ± 0.05 aB	24.35 ± 0.31 bB	20.99 ± 0.28 cC	23.28 ± 0.08 dB
ΔE (color change)	1	-	-	-	-
	7	12.29 ± 5.47	9.43 ± 3.44	11.66 ± 1.28	8.96 ± 2.45
	14	13.86 ± 1.74 a	8.36 ± 2.91 b	8.14 ± 2.11 b	9.19 ± 1.19 b

The results are the means of three replicates ± standard deviation, and the following abbreviations are used: C, control acid whey cheese; C + AP, cheese made with 3% of apple pomace AP; C + LL16, cheese made with 0.2% *L. lactis* LL16 strain biomass (100 g/0.2 g; C + LL16); C + AP + LL16, cheese made with 3% of apple pomace and 0.2% *L. lactis* LL16. The means with different uppercase letters (A–C) indicate significant differences (*p* < 0.05) between the storage days for each treatment. The means with different lowercase letters (a–d) indicate significant differences (*p* < 0.05) between the treatments at the same time interval (day).

## Data Availability

Data is contained within the article.
